# Why policy action should focus on the vulnerable commercial sex workers in Uganda during COVID-19 fight

**DOI:** 10.11604/pamj.supp.2020.35.2.24664

**Published:** 2020-07-06

**Authors:** Brenda Allen Kawala, Brian Kibiwott Kirui, Samuel Nambile Cumber

**Affiliations:** 1 Section for Epidemiology and Social Medicine, Department of Public Health, Institute of Medicine-Master in Global Health. The Sahlgrenska Academy at University of Gothenburg, Box 414, SE-405 Gothenburg, Sweden,; 2 Centre for Health Systems Research and Development, University of the Free State, Bloemfontein, South Africa,; 3 Office of the Dean, Faculty of Health Sciences, University of the Free State, Bloemfontein, South Africa,; 4 School of Health Systems and Public Health, Faculty of Health Sciences, University of Pretoria, Pretoria, South Africa,; 5 Institute of Health and Care Sciences, The Sahlgrenska Academy at University of Gothenburg, Gothenburg, Sweden

**Keywords:** Policy, vulnerable, commercial sex, COVID-19, Uganda

## To the Editors of the Panafrican Medical Journal

According to the Ugandan Ministry of Health, as of 24th June 2020, Uganda had registered a total of 797 cases of COVID-19 [1]. Two months prior, a majority of the positive cases in Uganda had been linked to being spread by long-distance truck drivers who drove into Uganda from Kenya and Tanzania. These neighboring countries had registered more COVID-19 infections and mortality. Seeing as the sex workers in Uganda are popularly found at border points where they interact with truck drivers [2,3] they were a key target in reducing community spread. In response, the Ministry of Health employed a self-proclaimed sex worker and Kampala city socialite to cast a commercial break advertisement as a health promotion measure. She warned girls at the Ugandan borders to avoid truck drivers to keep the nation free of COVID-19 [4]. This move went beyond the discrimination that sex workers in Uganda face to show the importance of such a marginalized group in the community spread of infectious diseases like COVID-19. Building from Uganda´s previous experience with HIV/AIDS, unempowered sex workers are at a higher risk for transmission with a 37% infection rate compared to 9% in the general population [5]. Some of these workers are sometimes forced to ignore regulations geared towards infection control as their ‘daily bread’ depend on the trade [6]. During the COVID-19 times, their plight is worsened by the inability to afford safe sex by using condoms and some of their customers preferring not to use protection [7]. Likewise, the majority of sex workers are illiterates as seen by only 53% of them having attained primary education in contrast to 73% in the general population [8]. Consequently, such vulnerable illiterate sex workers fail to fully comprehend the various health measures imposed by policymakers, and their bargaining power for safer sex is subdued.

Additionally, due to the cultural, legal, and social criminalization of the trade in Uganda, sex workers are not free to access social services required for them to have a safe sex life and they are often denied help [5,6]. This locks sex workers from potential benefits that white-collar workers could get from their employment. During the COVID-19 pandemic, sex workers in Uganda have been forced to undertake riskier behaviors that would possibly continue to dissipate community transmission of COVID-19 despite the total lockdown. The NGOs that provide free services are unable to reach them during a national lockdown as most sex workers resorted to working away from their usual stations. This grossly undermines the national intervention efforts to curb the pandemic. Lockdowns affected sex workers´ business sites such as bars, fishing sites, hotels, lodges, and night clubs. The busy streets in commercial areas where they would camp to wait for customers are devoid of people amid lockdowns and curfews. One woman narrated her ordeal which displayed risk-taking as she walks long distances to reach her customers at their places of convenience [6]. She does this despite the curfews and the risk of encountering the brutal police forces who enforce the lockdowns. The effects of COVID-19 in Uganda has exposed the inequalities and human right issues affecting criminalized communities such as sex workers. These include violence against women and engagement in risky behaviors as sex workers struggle to make a living [7]. Some sex workers are also being driven to extreme poverty as seen by a sex worker´s complaint about losing all her daily income of 50,000 Ugandan Shillings (13.5 USD) [7]. These workers´ strife for survival has forced some effort from the government such as food donations in one of the districts but this barely addresses their full predicament [9].

## Conclusion

The United Nations Development Program (UNDP) suggests three steps that can drive towards the inclusion of discriminated groups of people, in this case, the sex workers. As indicated above, they are serious foci for the continuous spread of infectious diseases such as COVID-19 in Uganda. The three steps include; (a) Examination into reasons that trigger discrimination, (b) Empowerment of the marginalized population and (c) Enacting reform policies nationwide that address the marginalized populations´ needs [10]. If Uganda can adopt such a framework, sex workers can become an important focus in public health interventions. Advocacy organizations are encouraging different lenses with which the public should view sex work as another form of labor with similarities to other types of jobs [8]. This can help reduce the stigma surrounding sex work, bring services closer to the more vulnerable populations as well as place sex work on the policy agenda. These organizations have also commenced initiatives directed towards empowering the most vulnerable sex workers both with knowledge and with tools that guide their trade.
